# Romanticizing decolonization and Asian epistemology: reflections on identity and space

**DOI:** 10.1007/s12564-023-09835-3

**Published:** 2023-03-06

**Authors:** Jack T. Lee

**Affiliations:** Moray House School of Education and Sport, University of Edinburgh, Edinburgh, UK

**Keywords:** Decolonization, Epistemology, Indigenous knowledge, Asia, Identity, Space, Globalization, Mobility

## Abstract

Recent calls for the decolonization of the academy demand recognition for diverse canons of knowledge . Asia’s economic ascent also imparts rising confidence among Asian scholars and institutions to promote indigenous knowledge. While these global calls for emancipation are invigorating, decolonial scholarship is prone to sterile theorization, historical fixity, and an overt romanticization of the Global South. Drawing on my lived experiences as an Asian academic, I reflect on decolonization and Asian epistemology from five different spaces in my life: (1) Northern Europe, (2) Toronto, (3) Southeast Asia, (4) Kazakhstan and (5) the United Kingdom. I analyze these spaces by using the concepts of intellectual captivity and decolonization from Syed Hussein Alatas and Kuan-Hsing Chen. Specifically, the tendency for decolonization movements to descend into nationalism, nativism, and civilizationalism provides provocative insights on epistemic justice (Chen, 2010). I demonstrate how epistemology as practice can reveal a colonial mindset even among academics who engage in social justice discourse and international work. I also highlight examples of indigenous knowledge that reinforce inequality based on race, gender, sexual orientation and religion. As more individuals with hybrid identities (race, culture, and nationality) enter academe and pursue careers that require international mobility, it is imperative that decolonization moves beyond reductive categories of identity that reproduce stereotypes. I conclude with reflections on the role of comparative and international education research in decolonization movements.

## Introduction

Recent calls for decolonization in the academy and the inclusion of diverse canons of knowledge have stimulated many lively discussions about epistemology and inequality (Marginson, 2021; Shahjahan, 2015). Contrary to the narrative that the academy upholds, knowledge production is hardly a neutral enterprise with universal metrics of excellence. Rather, Western epistemology and specifically Anglophones dominate global knowledge production. The timely calls for decolonization include incisive critiques against Western-centrism in my own field of comparative and international education, a sphere that is widely presumed to be progressive and cosmopolitan (Silova et al., 2017; Takayama et al., 2017). While these calls are intellectually stimulating and socially empowering in building solidarity among scholars, the arguments are prone to sterile theorization, historical fixity, and an overt romanticization of the Global South and indigenous knowledge. Discussions of decolonization demands scrutiny of behaviors and practices because knowledge operates beyond text and discourse. Everyday social interactions reflect deep rooted epistemology as individuals enact normative beliefs and ingrained worldviews. Moreover, the oft rehearsed condemnation against the Global North and white colonizers ignores many forms of subjugation enacted by the Global South and non-whites that are equally abhorrent if not worse. If social justice is truly the intellectual compass of decolonization, we must dismantle all forms of subjugation regardless of context or the identity of the oppressor.

This paper draws on my lived experiences as an academic of East Asian descent. My link with Asia remains eternal despite having left the region as a child. Regardless of my own cultural bricolage and affinity with borderlands (Anzaldúa, 1987), others continue to label me “Asian” through my race and professional work. International mobility has exposed me to different manifestations of decolonization and forced me to reflect on the politics of knowledge in the context of globalization. In this paper, I present five different spaces from my educational and professional history that prompted me to rethink decolonization and Asian epistemology: (1) Northern Europe, (2) Toronto, (3) Southeast Asia, (4) Kazakhstan, and (5) the UK. These examples are not demarcated by national borders because such a reductive framing cannot capture the complexity of social relations. Rather, *identity* and *space* mediate knowledge exchange and production more powerfully than the trappings of the nation-state.

The writing here is personal and experimental because I never intended to write about decolonization or publicize the incidents presented in this paper. The interactions disclosed in this paper are epiphanies that are commonly used in narrative research to illustrate changes in the narrator’s thinking (Bochner & Ellis, 1995; Denzin, 1989) and even existential crises in extreme situations (Zaner, 2004). While the paper exhibits some auto-ethnographic elements, my formal academic training is not in anthropology. However, recent sociopolitical turmoil in the UK and USA have prompted me to share these personal encounters and contribute to the discussion on decolonization and epistemic inequality. For example, Brexit has jolted British politics and spurred debates about British identity and insularism. The Black Lives Matter movement has challenged Britain to confront its complicity in the slave trade and led to calls for the removal of public statutes that celebrate colonial excess and the return of looted cultural artifacts that are currently housed in museums. The current British government agenda of “leveling up” (social mobility across the country) and widening access to education also highlight severe inequality due to class and race. In the USA, George Floyd’s violent death triggered nationwide protests over racial injustice and heated debates over America’s descent into populist politics and resurgence of white supremacy movements. The Covid-19 pandemic spurred anti-Asian rhetoric and even physical violence toward Asians in North America and the UK. These flashpoints raise serious questions about the liminal space between politics and education particularly when racialized educators and students feel unsafe.

My purpose in writing this paper is specifically three folds: (1) to build on the work of Syed Hussein Alatas and Kuan-Hsing Chen, as two giants in the development of social sciences in Asia, (2) to expose the contradictions between the rhetoric and the practices of decolonization and (3) to highlight the inequality that is promoted by some indigenous epistemology in Asia. The paper is structured largely in a chronological order starting with my experience in Northern Europe in my twenties and concluding with my present life in the UK. Recent events remain more visceral; therefore, the paper does not provide equal coverage of each of the five spaces but rather resembles a chromatography of memory with striking bands concentrated at the final two spaces (Kazakhstan and the UK). The incidents presented in this paper would be impossible to document through any planned research study—not simply due to a coverage of over two decades in time but also access to spontaneous social interactions in unexpected settings.

## Decolonization and Asia

This paper draws on the ideas of two Asian intellectuals at the forefront of decolonization: the late Malaysian sociologist and politician Syed Hussein Alatas (1928–2007) and contemporary Taiwanese scholar Kuan-Hsing Chen. In the 1970s, Alatas argued for a more nuanced view of decolonization that extend beyond normative political and economic analyses to *intellectual imperialism*. Alatas’s rendering of Western domination emphasized exploitation, tutelage, and conformity as some of the key operational mechanisms (Alatas, 2000). His critique was not simply a tirade against the West, but it was also a caustic appraisal of *intellectual captivity* among subjugated nations in the Global South (Alatas, 1977b). In particular, he noted the complicity of the ruling elites in the Global South:

This whole phenomenon of uncritical transmission of thought can be regarded as unconscious continuation of colonialism not in the political but in the cultural sense. (Alatas, 1956)

This emphasis on cultural mores also appears later in Chen’s masterpiece *Asia as Method* (2010). The critique of cultural imperialism may seem very quotidian today; however, the patterns of exploitation and obsequious mimicry of the West continues to persist (Bhabha, 1994; Nandy, 1983). Despite Alatas’s intellectual contributions and forays into pro-independence politics in Malaysia, he never advocated a radical de-linking from the West. Instead, he argued,

I am not suggesting that we should close our minds to genuine knowledge from any part of the world. We should assimilate as much as possible from all sources, from all parts of the world, all useful knowledge... We should assimilate whatever is necessary for progress. We should be practical and independent, and at the same time tap the maximum from our own tradition. (Alatas, 2000, p. 27)

This pragmatic approach to epistemology set Alatas apart from other decolonial thinkers who called for severing ties with the West to rectify “under development” in the Global South (e.g., Gunder Frank, Immanuel Wallerstein, and related neo-Marxist dependency theorists in Latin America). Acolytes of dependency theory and world-systems theory extended de-linking as an economic concept and suggested the rejection of Western epistemology for bona fide independence. Admittedly, Alatas’s view of colonialism and neo-colonialism was very much bounded by the geography of Asia and the politics of his time. His clarion calls targeted largely Asian social scientists working in Asia rather than diasporic scholars of Asian heritage or those with hybrid cultural and racial identities—this omission is noticeable when comparing his writing to more recent scholarship on decolonization.

Nearly half a century after Alatas’s exposition, Kuan-Hsing Chen echoed similar sentiments in another intellectual awakening for Asia. In *Asia as Method* (2010), Chen admonishes post-colonial scholarship for its obsession with the West as a reference point. In his view, decolonization movements often adopt three regressive stances when calling for emancipation: (1) nationalism, (2) nativism and (3) civilizationalism. These stances will be explained later when making sense of my lived experiences. Chen’s concept of *geo-colonial historical materialism* views Asia as an actor in history rather than merely as a bystander who witnesses and experiences actions by the West. He simultaneously challenges colonialism, imperialism, and Cold War subjugation. Chen does not simply confront decolonization from his field of cultural studies, but he also integrates ideas from psychoanalysis and radical geography to provide insights on emotions, identities, and space. The formidable interdisciplinary nature of his work extends decolonial scholarship beyond the normative critiques from Marxist and critical race scholars. Socialist regimes and Asian states have also maintained brutal colonial machineries as the Soviet and Japanese empires have demonstrated in history. Similarly, recent Russian aggression in Georgia, Syria, and Ukraine and Chinese involvement in Africa's development (King, 2013) reflect power contestation and subjugation  rather than colonial ambitions.

Strikingly, Chen’s interpretation of “Asia” extends beyond geography to include history, politics, cultural representation and emotion. He advocates studies that are not constrained by the nation-state—a Westphalian framing that echoes the methodological nationalism critique from comparative education scholars (Dale & Robertson, 2009; Shahjahan & Kezar, 2013). For Chen, decolonization is not merely about national independence movements or knowledge contestations, but it must also examine culture, mind, desire and body (Chen, 2010, p. x). It other words, decolonization is about “action, subjectivity, thought, cultural forms of expression, social institutions and global political and economic structures” (Chen, 2010, p. 112). He encourages inquiries into emotions:

…rather than equivocating about or suppressing the emotional conditions of the subject, I have found that critical cultural studies works best when it brings sentiment to the forefront, making it a source of thought and analysis. (Chen, 2010, p. xvi)

This emphasis on the affective dimension of coloniality (Maldonado-Torres, 2007) is also evident in Alatas’s extensive scholarship. Echoing Alatas, Chen points out that scholars in Asia have yet to decolonize scholarship and knowledge production particularly given the aggregate effects of colonialism, imperialism, and the Cold War. In short, Chen dramatically extends Alatas’s thesis on colonialism by taking a wider recognition of hegemony and engaging explicitly with globalization.

## East Asian roots

Some biographic disclosures are necessary to provide a context for my reflections and critiques which appear later in this paper. Border crossings and intercultural existence have been constants in my life through fortuitous developments rather than deliberate planning. I was born in Taiwan and educated in its public school system until age 10. Through immigration, I arrived in the USA as an ESL (English as a Second Language) student and completed the remainder of my compulsory education there. I received all my higher education in Canada (bachelor, master’s, PhD). Today I identify as a Canadian or more specifically Taiwanese-Canadian after 20+ years of living, studying and working in Canada. However, this period has been interrupted by several sojourns outside Canada as this paper will illustrate. I have lived and worked outside Canada since 2014. I currently live in Scotland as my 8th country of residence. I am separated from colonialism by one generation. My grandparents grew up under Japanese occupation in Taiwan and spoke fluent Japanese before having to learn Mandarin as adults when the Japanese Empire collapsed at the end of World War II. Japanese colonialism in Taiwan remained a mystery to me until my adulthood because neither did my American education nor Taiwanese relatives delve into this history. Instead, Japanese colonialism and culture remain largely vaunted in Taiwanese society as people ascribe very positive traits to both: an organized society, rapid modernization, admirable work ethics, precision engineering, and impeccable social etiquette. Surprisingly, this infatuation cuts across age, gender, and class. Colonialism may be distant and abstract to Taiwan’s younger generation, but the older generation often reminisces about the colonial era despite the brutality of Japanese occupation.

## Confronting race in Northern Europe

My exposure to Europe began with living in Germany and Norway in my 20s and 30s, respectively. Europe was a fabled land that dominated my American education through literature and history classes. As I prepared to move to Germany in 1998 to start an internship at a research lab in Potsdam, I was alarmed by news stories of neo-Nazis attacking foreigners. When asked, my German colleagues dismissed these incidents as fringe activities and assured me the city was very safe. I later moved to a post at a Max Planck Institute in Berlin and rented a flat in the former Eastern half of the city. On one visit to the hinterland of former East Berlin, my Greek colleague joked that I should lock the car door on my side given my conspicuous *mandelaugen* (almond eyes, a derogatory term for Asians) in a neighborhood known for right-wing movements. Paranoia became a reality when I later noticed neo-Nazis on the subway on a regular basis and witnessed a few of them frequenting a building near my flat. Unimaginable for me at the time, my race became a liability for personal safety.

In my interactions with Germans and expatriates in Germany, it was quickly apparent that my skin color and my command of English generated cognitive dissonance. Strangers often interrupted me in mid-sentence to ask my country of origin. Identifying simply as a Canadian was not an acceptable answer. The jarring reactions I received in Germany were also common when I lived in Norway in my thirties as an exchange student. During roll call on the first day of class at the University of Oslo, a Norwegian professor who is internationally renowned for research on the Global South^1^ asked smugly, “Jack Lee? My class list shows you’re from Canada, but surely you must be Chinese, right?!” My explanation about being born in Taiwan and subsequent immigration to Canada came across like an apology for self-identification. The class was subjected to another apology when the professor asked a student why she identified as Norwegian on paper when she clearly “did not look Norwegian.” The entire class listened to her personal history of being born to an Afghan father and Norwegian mother, raised fully in Norway, and educated in Norwegian her entire life. These public inquisitions struck me as both intrusive and anachronistic in reducing individuals to their racial identities and questioning their belonging. Even my Norwegian classmates who were well immersed in intercultural and international activities were puzzled by my fluency in English and insisted on the full truth. My reply to these queries about identity and language has always been that anyone growing up in an education system would naturally be fluent in the language of instruction. Would Europeans comment on a white Canadian’s command of English? Through these encounters, I often wondered who could credibly identify as “Canadian” if citizenship, education, and work experience over two decades did not suffice. I eventually refused to explain my identity and family history to strangers because such interactions were both exhausting and demeaning. On a more egregious level, such interactions perpetuate a narrative of a monolithic white North America where racial minorities have “illegitimate” claims on belonging. On one occasion at the Munich airport, a security guard glanced at my Canadian passport, conducted a body search, and insisted on guessing my country of origin. I retorted, “Simply Canadian!” and walked off to catch my flight.

Race became an overwhelming part of my identity in my Northern European experience while discussions of epistemology and decolonization were distant abstractions. Chen points out that “the cultural imaginary is disseminated to different social fields, shaping the imaginations of both colonizing and colonized subjects” (Chen, 2010, p. 111). My educational attainment perhaps afforded some measure of social acceptance. At dinner parties in Norway, it was not uncommon to hear xenophobic sentiments leveled against the country’s established Pakistani community (people who arrived in the 1970s as guest workers and raised children fully in Norway) and its emerging Somalian community. Ironically, these sentiments were flaunted in front of the expatriate guests. The non-whites were exempt from these critiques perhaps due to our social capital among Norwegian friends.

My experience in Northern Europe surprised me in many ways because I grew up viewing Europe as a utopia. Germany and Norway are often touted today as European beacons of social democratic values, humanitarian generosity and economic prowess. However, the discourse of multiculturalism and race in both countries lag several decades behind North America’s. Granted, racism and bigotry are also rampant in the USA and Canada, but the low level of consciousness in Germany and Norway was striking even among the intelligentsia. In 2019, a senior Norwegian university leader known for work on internationalizing education and advising the Norwegian government reminded me over lunch, “I know you’re Canadian, but your English has a slight Asian accent.” I refrained from replying that his Norwegian accent was surprisingly thick despite building a successful career in international education. While national pride was palpable in Berlin in the late 1990s as the city transformed to reclaim its capital status again, Germans were mostly welcoming and optimistic rather than exclusionary in the form of ethnic nationalism. Chen’s thesis that nationalism often follows colonialism (or more accurately de-Cold War in Germany and Norwegian independence from Sweden) did not fully play out in my experiences in both countries. Rather than ethnic nationalism, the prevailing climate was racial essentialism. In both countries, alternative epistemologies were rarely discussed even among elites who frequented transnational and intercultural spaces. My vision of Germany and Norway as utopias disintegrated very quickly when rudimentary understandings of race dominated social interactions.

## Awakening in Toronto

The years I spent in Toronto to complete my doctoral studies were memorable and empowering in many ways. While this city cannot compare to New York City or London in scale, Toronto is unequivocally Canada’s most diverse and lively metropolis. I left Vancouver’s strong pivot toward Asia to discover Toronto’s impressive cosmopolitanism. My academic and social ties extended far beyond what I could ever imagine from traveling or reading. I lived in Little Portugal, moved to Roncesvalles (Polish/Lithuanian/Ukrainian community), socialized in Koreatown and attended the beehive of events organized by the Comparative, International, and Development Education Centre and the Munk Centre of Global Affairs. For the first time in my life, I was exposed to a scholarly examination of Asian epistemology in all its diverse forms. This inclusion was particularly palpable in Ruth Hayhoe’s classes and thesis group as she encouraged deep inquiries that highlighted the tremendous intellectual contributions of many Asian societies. From indigenous higher education in northern Canada to international partnerships in Cambodia and the development of an open university in India, students contributed to lively discussions on theories and research design. Hayhoe’s own understanding of Chinese culture, Confucian philosophy and Asian epistemology was extraordinary and inspiring for those of us who benefited from her supervision and mentorship. On the surface, Hayhoe’s own work reflects the civilizationalism that Chen points out as a feature of decolonization when knowledge contestation draws on deep intellectual veins across an entire civilization (e.g., Sinic, Western, and Persian). Seminal works by Francis Fukuyama (*The End of History and the Last Man*, 1992) and Samuel Huntington (*The Clash of Civilization*s, 1996) exemplify a civilizational approach to understanding global politics. More recently, neo-Confucian scholars who link state ideology in East Asia with Confucian thought also reflect the power of civilizationalism in explaining the roots of social development (Chen, 2010). However, a closer reading of Hayhoe’s prolific work reveals that her doctrine is genuinely about intellectual synthesis and knowledge exchange rather than contestation or hierarchy building. Her writing echoes Alatas’s pragmatism while integrating the contributions of indigenous knowledge. Hayhoe’s work does not exude the triumphalism or fatalism that is evident in Fukuyama and Huntington’s assessments of history, Western liberalism, and cultural conflicts. In exposing the shortcomings of nativism, nationalism, and civilizationalism as misguided decolonization, Chen advocates critical syncretism “to avoid reproducing colonialism and to go beyond the politics of resentment that bind colonizer and colonized together” (Chen, 2010, p. 72). Hayhoe’s extensive writing on Chinese higher education and comparative education strongly reflects the intellectual openness and agency in Chen’s critical syncretism as she argues tirelessly for cultural exchange and epistemic synthesis.

In retrospect, the spaces for knowledge production in Toronto were possible due to continuous institutional support and individual commitments rather than the ethos of a multicultural city. Undoubtedly, not all spaces within the University of Toronto were so inclusive, and certainly not all doctoral students were exposed to Southern epistemology. Therefore, our understanding of knowledge production should not assume that certain types of geography (Global North) or identity (white Westerners) are inherently incompatible to intellectual decolonization. Racial or cultural identity alone does not automatically trigger decolonial thinking as Alatas explicated at length in the 1970s. Anzaldúa (1987) later emphasized the need to work with white allies in challenging racist thinking.

## Understanding post-colonial Southeast Asia

My doctoral work in East and Southeast Asia stimulated my intellectual growth in immeasurable ways. I lived in Malaysia, Singapore, and Hong Kong for six months as a peripatetic researcher. British colonialism remained a strong feature in all three societies despite the bygone days of colonial occupation. Many of the policymakers and institutional leaders I interviewed held advanced degrees from the UK. Many of the universities I visited had strong links to British institutions. I came to appreciate Malaysians’ hospitality and seamless navigation between very different cultures: Malay, Chinese, Indian, and remnants of British. Ethnic relations in both Malaysia and Singapore provided a valuable lesson on multiculturalism in a post-colonial context. Affirmative action in Malaysia rectified generations of inequality; yet, it also persisted as an instrument of segregation. Ethnic Chinese and Indians continue to be excluded from many pathways of social mobility (e.g., restricted access to public universities and civil servant posts). Rising consciousness of Malay culture included the emergence of an impressive Islamic finance sector that forbade speculative banking practices because community interests superseded personal profit seeking. Yet, this ardent embrace of Malay culture also fueled religious fundamentalism and ethnic nationalism in both social spaces and political discourse. Chen’s critique of nativism and nationalism as common features of decolonization were fully evident in Malaysia. “Nativism brought people’s focus from the imperial centers back to their own living environments; in the process of reclaiming tradition, it tilted the balance away from the previous, sometimes worshipful embrace of the modern” (Chen, 2010, p. 81).

While Hong Kong did not exhibit such ethnic tensions, its rampant use of domestic workers from Southeast Asia raised questions about a different kind of subjugation. Every Sunday during my stay, a public ritual unfolded in central Hong Kong like clockwork: a sea of domestic workers emerged to enjoy reprieve from their indoor work. This ritual did not involve interactions with Hong Kongers, but instead it was a conspicuous segregation based on class and race. The sight of these migrants occupying every corner of public space to socialize among themselves was a jarring contrast to the city’s soaring skyscrapers and ostentatious openness to the world. Similarly, Singapore’s vaunted modernity was also difficult to reconcile with its foreboding climate of censorship, heavy reliance on domestic workers, and rising social inequality. While meritocracy is a pillar of Singaporean identity, the elites are noticeably ethnic Chinese. These critical observations may stem from my standpoint as an outsider to all three societies; however, local activists and community workers are fully cognizant of these inequalities. Accepted norms of labor division, social relations, and space segregation reflect a disturbing epistemology of inequality that is rarely discussed in decolonization efforts. Southeast Asia and Hong Kong have certainly produced their share of critical post-colonial scholarship, but scholars seem to avoid looking into mirrors that might implicate indigenous norms and values for subjugation on multiple levels.

Civilizationalism is also particularly noticeable in Hong Kong and Singapore in their celebrated branding as East–West gateways. Countless marketing slogans and policies (including education) exploit this ubiquitous trope to engage with outsiders and attract international partnerships. While this approach does not pit one civilization against another as Chen points out in some decolonization movements, this framing does reinforce crude interpretations of civilization in a post-colonial space. A space that is simultaneously East and West can sidestep questions about identity because multiple subjectivities are assumed to thrive in such an environment. Interestingly, the celebrated free marketplace of Hong Kong and Singapore replicates a free cultural space (at least in discourse). In short, decolonization seems unnecessary if cultural and epistemic borders are blurred.

## Witnessing independent Kazakhstan

Following the completion of my PhD, I moved to Kazakhstan to work as an assistant professor at Nazarbayev University, a new university that shouldered many ambitious national reforms: international engagement, economic transformation, innovation and modernization. Specifically, the country’s reliance on oil and gas as well as on foreign universities to educate its talented youth abroad was untenable. Starting in the early 1990s, millions have been spent sending over 10,000 Kazakhstani students overseas to pursue full degrees at leading universities (Bolashak Programme). The prospect of working at a new university with an explicit agenda on capacity building and internationalization was very enticing given my own research focus on international higher education and the politics of education. Moreover, the opportunity to live in a part of Asia that I knew very little about was exciting. I arrived in 2014 and immediately started private lessons to learn Russian given its widespread usage in northern Kazakhstan, where I lived. The vast majority of the foreigners working at Nazarbayev University eschewed language education and lived in a bubble that was largely disconnected from the local community—this was not the lifestyle I envisioned for myself. Instead, I persisted with my weekly Russian lessons for my entire stay in Kazakhstan (2014–2017), scoured local markets, frequented local art performances, traveled across the country, and swam in − 20C during the annual Epiphany (Кpeщeниe) to understand this country and Central Asia.

During my time in Kazakhstan, the state implemented countless reforms to improve its education system (e.g., research capacity building, internationalization, quality assurance, teacher training). Noticeably, the drive to revive indigenous epistemology, particularly the use of the Kazakh language, was remarkable as the country approached 30 years of independence. Today, all students across the country are required to take Kazakh language courses even at Nazarbayev University, where the language of instruction is English. I found these developments inspiring and far more visionary than places like Taiwan, Singapore, and Hong Kong, where local languages are completely expunged from the formal curriculum without much consciousness among educators or parents. I marveled at Kazakhstan’s foresight and fortitude in leveraging policy to cultivate its indigenous language, arts, and literature. However, the daily lived experiences presented many junctures of exclusion that are rarely discussed due to social and political sensitivity in Kazakhstan. For example, some ethnic Russian students in my class objected to a proposal by their classmates to use the Kazakh language exclusively in their social media interactions (e.g., in a WhatsApp chat group created by students). These minority students along with Russian-speaking Kazakhs were berated by their peers for not using Kazakh on social media and not speaking Kazakh well. Students also shared stories of being tested for Kazakh fluency in the middle of interviews for jobs and program admission even though the selection criteria never listed Kazakh fluency as a requirement. These practices excluded ethnic Kazakhs, Russians, Ukrainians, and Germans who grew up in Kazakhstan schooled in the Russian language. At one point, students even confronted me on my decision to learn Russian rather than Kazakh. At a meeting at the Ministry of Education and Science to review the progress of our state-funded project, the chairwoman loudly admonished my Kazakh colleague for speaking English to me and forbade further translations.

Indigenous revival extended far beyond language and epistemology in Kazakhstan. In one social media post, I questioned the value of a local research project that developed a garbage bin which acknowledged users with an audible “thank you.” My post elicited severe censure for belittling research that was conducted by locals. Strong xenophobic reactions escalated to phone calls from the public to the university demanding to know why a foreigner was hired to be a professor. These comments echoed the complaints I heard regularly from taxi drivers: *why do we need foreigners working in our universities?!* On several occasions, drivers assumed I did not understand any Russian and proceeded to lecture my local colleague in the taxi on the virtues of Kazakh nationalism. A national university should absolutely employ and cultivate the nation’s eminent scholars and contribute to social progress. However, no leading university in the world hires exclusively from the local workforce.

As Chen points out, nativism and ethnic nationalism are often inseparable as reactive and pernicious forms of decolonization. Specifically, nativism is “expressed in the xenophobia of the colonized—is indeed a return to colonial racism” (Chen, 2010, p. 86). The prominent rhetoric of modernization and international engagement in Kazakhstan masks a rising tide of ethnic nationalism and insular epistemology. Conflating indigenous empowerment with the absolute rejection of all other forms of knowledge debases intellectual and social development (Alatas, 2010; Hountondji, 1995) such that decolonization becomes “uncritically supportive of the ethnocentric nation-building project” (Chen, 2010, p. 65). Moreover, decolonization or specifically de-Sovietization in Kazakhstan requires a much more substantial effort if the country is serious about its autonomy. Kazakhstan continues to replicate Russian norms through its penchant for strongman governance, authority, bureaucracy and positivist ontology. Kazakhstan’s reliance on the Russian economy and Russian military for national prosperity and security reveal enduring imperialist links.^2^ Civilizationalism may also partly explain decolonization in Kazakhstan because the country’s engagement with the Russo-sphere (Russia and post-Soviet states) generally persists without challenge; however, engagements with the West and China often trigger skepticism or uproar when deemed as encounters with a fundamentally different civilization that threatens a Slavic or Turkic civilization.

In Kazakhstan and many other parts of Asia, indigenous epistemology also celebrates patriarchy and reinforces gender inequality; yet, scholars of decolonization often ignore these objectionable norms. Many Confucian societies continue to promote archaic gender roles. My own homeland of Taiwan continues to endorse a nuclear family template with limited recognition for individual identities and aspirations. Women’s high attainment of education in places like Taiwan, China, Korea, and Japan has not translated into progressive social attitudes that challenge patriarchy. In Kazakhstan, a European colleague visited a local pre-school that he was considering for his son. In one exercise to learn vocabulary and analytical skills, children were asked to review images depicted on multiple cards and identify the single card that did not belong (e.g., a card showing an apple among many others showing modes of transportation). When given multiple cards showing women engaged in different activities (cooking, caring, working, shopping), many children were perplexed. A few children arbitrarily picked a card, but the teacher gently corrected their “mistakes” and selected the card with a woman in formal attire in a professional setting as the one that did not belong. This colleague never took his son back to this pre-school again.

The indoctrination of gender stereotypes in Kazakhstan extends from an early age well into adulthood. International Women’s Day is a widely celebrated holiday in post-Soviet states. This very public recognition of women’s contributions in society is remarkable as a nation-wide effort. However, lived experiences present numerous contradictions again. On one International Women’s Day, my university distributed a mass email thanking our women staff for their academic and administrative work. But this seemingly innocuous message came attached with a prominent image of a pregnant woman. Several staff members, particularly among the expatriate community, protested that the image reinforced a sexist stereotype that reduced women to their reproductive role. In my department’s own celebratory dinner to recognize our women colleagues, the obligatory toasts from local male colleagues repeatedly emphasized women’s physical beauty, kindness, and emotional intelligence. Ironically, several women held positions of authority within our department; yet few men celebrated their leadership skills or managerial acumen. In subsequent years, I avoided such dinners to distant myself from retrograde gender awareness. One year I celebrated this day in a restaurant outside Kazakhstan with a few Central Asian women colleagues as a memorable respite from the conference we were attending.

Despite my estrangement from the gender discourse in Kazakhstan, the reality of gender relations was impossible to avoid. Many of the top students in my classes often sought advice on pursuing PhDs in the future. Many of these bright, enthusiastic individuals were women. In long discussions to plan their futures, many of these women confided that they faced tremendous resistance from two powerful individuals when considering further education: the husband (or boyfriend) and the mother-in-law. Some mothers-in-law rebuked, “You have already wasted so much time studying for a master’s degree! Why do you need a PhD?!” In a workshop to advise our students with doctoral aspirations, a Kazakh colleague strongly advised the roomful of women, “Never finish a PhD before getting married because few men would want to marry a highly educated woman! Make sure you find a husband before defending your thesis!” This searing warning came not from a male colleague but rather a woman who conducts research on gender inequality. Throughout my experiences in Asia, the blatant sexism and misogyny I witnessed forced me to reflect on the limits of nativism when indigenous revival in fact reinforced gender inequality.

Related to gender inequality is the pervasive heteronormativity in many Asian societies. Gender as a discourse is often reduced to issues that affect heterosexual women and sometimes even explicitly about married women only (e.g., policies and research on the experiences of women in balancing work and family life). Single women might receive some attention in the literature, but divorced women, widowed women, rural women, and LGBTQ women are largely absent from the gender discourse. Given the taboo nature of sex and the religious orientations of some countries, homosexuality is not openly discussed in most settings. Again, it might be convenient to dismiss these concerns as issues that only apply to conservative Muslim societies such as Malaysia, Indonesia, or Kazakhstan. However, even in a place like Taiwan, which is renowned for its progressive social values and trailblazing status as the first country in Asia to legalize same-sex marriage, heteronormativity is overwhelming in social settings. Intrusive personal questions about marital status and children are not uncommon in professional settings in many parts of Asia. Even Taiwan’s incumbent president, Tsai Ing-wen, faced questions and criticisms about her decision to remain unmarried and childless as the first woman president in Taiwanese history.

## Surviving the British Empire

In 2017, I left Kazakhstan and began working in England. Having been schooled on Britain’s contributions to higher education as a comparative education student, I looked forward to experiencing its higher education first-hand. Britain’s extensive history in comparative education and its large community of social scientists were attractive factors. The thought of living in the heart of a former global empire did not concern me given these professional considerations. While I was prepared for the dramatic cultural shift from Kazakhstan to Britain, I was not prepared for British centrism or exceptionalism. At my first conference in the UK, a senior British scholar asked how I was enjoying the event. I naively replied that the quality of presentations and papers was quite high, but the topics were noticeably very British both in scope and theoretical framing. My comments elicited a defiant retort, “Very British?! But rightly so, as it should be!” This reaction surprised me given that the scholar was known for research on international education as well as the Global South.

In preparing to write this paper, I went out and bought a copy of Kuan-Hsing Chen’s *Asia as Method*. A few colleagues had actually recommended this book to me more than a year ago, but my deep skepticism of geography being deployed as a methodology and weariness towards acerbic anti-Western critiques deterred me from locating a copy. To my genuine surprise, I found Chen’s arguments cogent and powerful. His deft assessment of post-colonial discourse and Asia’s nascent decolonization movement raises many important questions about complicity and multiple forms of subjugation. I quickly shared some positive reflections about this book with a British colleague who regularly advocates decolonization. To my astonishment, this colleague quipped, “Sorry to burst your bubble and expose your echo chamber, but no one has mentioned that book in my field. The disinterest and disdain in the subaltern fortified the perimeter of knowledge: the self as the arbiter of legitimate epistemology. Another example took place at a research training session in a Russell Group institution a few years earlier. An esteemed British professor proceeded to teach a roomful of academics from Kazakhstan how to conduct a literature review even though many of us completed PhDs in the West. He then mocked variants of English outside the British Isles as inferior imitations especially among North American speakers. During coffee break, I informed the organizers that most of us were already teaching our own students how to conduct literature reviews, and we certainly did not fly thousands of kilometers at a significant cost to our employer both in time and money just to learn how to write a literature review. The agenda was quickly modified for the remainder of the training program. Most alarmingly, these examples of epistemic erasure do not come from academics who focus on the UK research topics or navigate provincial circles. Rather, the examples come from individuals who specialize in the Global South, advocate social justice, pursue international projects, and supervise many international students. Is decolonization and the broader project of social justice merely a performative exercise while attitudes and personal behaviors remain staunchly British and ethnocentric?

In my interactions with British colleagues, British centrism and exceptionalism continue to catch me off guard in many different situations: a colleague explaining to me the basics of student assessment despite my two decades working in higher education; a program administrator correcting my pronunciation in front of a roomful of students; a department head reminding me that I lack experience in academic leadership despite having spent a decade leading institutional initiatives in Canada and more years contributing to institution building in Kazakhstan (e.g., faculty senate, committee chairs, and research ethics board). Seemingly, experiences gained outside the UK are irrelevant even though the bulk of my professional and academic work was done in Canada at two prominent universities (University of British Columbia and University of Toronto). At one job talk in England, a hiring panel was baffled by my project on Southeast Asian higher education. The chair of the panel asked with disbelief, “Why do you call this a higher education research project if you’re using theories from political science?” Exclusion obviously operates on multiple fronts: race, language, geography, and theoretical framing. Chen (2010) has written eloquently about the psychology of colonization which views people from the colonies as immature and deficient in development and requiring assistance from the colonial master to attain maturity. Alatas (1977a) wrote extensively about the negative images of colonial subjects among British eyes. The colonial mentality views imperialism as a “necessary stage in human progress” (Alatas, 2000, p. 24). In short, Western experiences become enshrined as rites of passage for academics despite the rhetoric of decolonization and workplace inclusion—this bias persists in both the Global North and the Global South when universities and employers equate experience in the West with professional achievement. The persistent “othering” through social interactions in Britain reinforces cultural and epistemic borders even if I do not consciously subscribe to these borders in my self-identification. In other words, while I am not always aware of my accent, cultural norms and professional history, British colleagues habitually point out my deviations from British norms and even venture to impart advice to address perceived deficiencies. To what extent is international and intercultural experience actually valued in the daily routines of academia? What constitutes legitimate knowledge and experience? Who are the arbiters? Edward Said’s *Orientalism* (1978) is alive and thriving at the heart of the empire and the halls of academia even nearly half a century after the publication of his magnum opus.

## Implications for decolonization and comparative education

Building on Alatas and Chen’s work on coloniality, I concur with their arguments that decolonization must include indigenous epistemology. Significant scholarship dating back to the 1950s exists on this line on of argument; therefore, I will not belabor this point. Unfortunately, many decolonial scholars and activists continue to view different forms of epistemology as mutually exclusive—a contestation of knowledge that replicates realist thinking in international relations. Much of the warfare in human history is underpinned by realist concerns over power, territories, and resources, yet this conceptual framework circulates in decolonial circles without much reproach. This antiquated view of knowledge is unhelpful for intellectual progress and prone to the nativism, nationalism, and civilizationalism Chen cautioned against. Ghanaian philosopher Kwasi Wiredu warned that African philosophy must not reject modern logic and epistemology as “un-African” and “content ourselves with repeating the proverbs and folk conceptions of our forefathers” (Wiredu, 1980, p. x). Likewise, Beninese philosopher Paulin Hountondji called for “intellectual responsibility” in indigenous research to avoid a static view of African cultures (Hountondji, 1996). Raewyn Connell’s volume, *Southern Theory*, provides thought-provoking deliberations on indigenous knowledge and the development of social sciences in the Global South. Decolonization must be integrative in its assemblage of epistemology rather than exclusive or additive. Although Chen is skeptical about cosmopolitanism as an intellectual foundation, a cosmopolitan approach to knowledge is far more promising than deference to indigeneity. Decolonial scholarship has provided blistering critiques of Western hegemony, but it has yet to confront the shadows of indigeneity to truly provide a transformative intellectual project. As an interdisciplinary field with diverse historical veins, comparative education is well positioned to test the limits of cosmopolitanism and examine indigenous knowledge closely.

While Chen has provided some action steps for “Asia as method,” I argue that his framing occasionally reverts to a geographic definition of Asia that echoes Alatas’s work rather than embraces an ideational conception that accounts for personal subjectivities. For example, Chen differentiates those living inside and outside colonial territories: "Unlike those living in the imperial centers, our own existence is internal to the decolonization movement.” (Chen, 2010, p. 81). This strange binary view reduces space to geography and assumes subjectivities. Diasporic Asian scholars such as myself or Asian scholars born and raised in the West do not figure prominently in Chen’s analysis; yet we face the same colonial attitudes and discriminations, if not more frequently, than our counterparts living in Asia. Decolonization is not a movement confined to former colonial territories or colonial subjects. Ashis Nandy said it best: “The West is now everywhere, within the West and outside; in structures and in minds” (Nandy, 1983, p.11). Mixed-race scholars also represent an important demographic segment that is rarely discussed in decolonial work. As Anzaldua noted, “Living in a state of psychic unrest, in a Borderland, is what makes poets write and artists create” (Anzaldúa, 1987, p. 73). Whether it is Asians in the West, Westerners in Asia, or multiracial academics, these individuals are increasingly common under the hyper mobility that characterizes academic careers today (Collins & Ho, 2018; Lee & Kuzhabekova, 2018). Extended sojourns such as full-time employment and immigration can expose social scientists to diverse epistemologies. In fact, the field of comparative education was created by many immigrant and bi-cultural scholars who personally experienced different education systems. My own experience as a student in Taiwan, US, Canada, and Norway exposed me to fundamentally different norms and aspirations in education. Consequently, a geographic conception of Asia is inadequate for understanding the pluralism in identities and mobilities. In operationalizing Chen’s concepts, Jane Kenway (2015) explicitly calls on Asian and non-Asian scholars as well as scholars located outside the Asia region for collective action on decolonization. Many more empirical studies are needed to truly explicate the roles of identity and space in epistemic transformations and decolonization.

Like Chen, I draw attention to the sharp inequalities that are reproduced by indigenous epistemology. If decolonization is truly about confronting subjugation, why should indigenous knowledge be exempt? This paper has presented many forms of subjugation based on lived experiences. The prospect of decolonization replacing one form of subjugation (coloniality) with another form (patriarchy, racism, xenophobia, homophobia, and religious fundamentalism) is alarming. Seemingly, indigenous knowledge is assumed to be empowering and immaculate. Perhaps the climate of political correctness has elevated indigenous knowledge to an intellectual sanctum and discouraged academics from critically evaluating its role in achieving social justice.

## Conclusion

In assembling the personal experiences presented in this paper, my objective is not to make summative judgements about these cultures or spaces. I view culture as a dynamic terrain that continuously shifts with time and space. My purpose here is to highlight *epistemology as practice* rather than epistemology as an abstract matter confined to texts and discourses. I have used narrative as a methodological device in this paper to highlight identity and space in decolonization (Ellis, 2002). Personal stories allow *witnessing* (Denzin, 2004; Ellis & Bochner, 2006), which is not readily available via other research traditions. Syed Hussein Alatas and Kuan-Hsing Chen’s calls for introspection plus recent political events (i.e., Black Lives Matter, Brexit, COVID-19 pandemic) prompted me to write this paper. As Chen encouraged, I have highlighted some of the complex links between history, geography, and knowledge as well as the contradictions of decolonialization. Proponents of social justice and decolonization may retain extremely colonial attitudes or essentialist thinking that contradict their rhetoric. Researchers whose careers are built on projects in the Global South may in fact espouse a deficit view of the subaltern that continues to echo Edward Said’s orientalism. Furthermore, efforts to decolonize can mask deep inequalities that are inherent in indigenous epistemology. If decolonization is truly about confronting subjugation, critical assessments of all forms of knowledge and practice are essential for emancipation. An integration of diverse epistemologies is a productive way forward rather than an exclusive or additive approach that amplifies the *politics of resentment* (Chen, 2010). This paper is written with the hope of stimulating further discussions on decolonization and creating authentic inclusions in academic spaces.

## Footnotes

1 My use of the term “Global South” in this paper is not to suggest expertise on a wide swath of the world or homogeneity of a region but rather to anonymize personal identifiers. Similarly, the term “colleague” is use very loosely in this paper to denote a professional contact rather than specifically a person working at my institution or in my field.

2 A recent example is the nation-wide protest in January 2022, which was the largest political unrest in Kazakhstan since the country gained independence in 1991. The Kazakhstani government quickly requested Russian military intervention to restore order.
